# Clinical characteristics and predictive indictors of macrolide-unresponsive *Mycoplasma pneumoniae* pneumonia in children: a retrospective study

**DOI:** 10.3389/fped.2024.1489389

**Published:** 2024-12-03

**Authors:** Yun Li, Yunwei Liu, Xinying Chen, Xiaolan Xiao, Yiting Chen, Lianyu Wang, Wenwen Jiang, Jinghua Yang

**Affiliations:** ^1^ Department of Pediatrics, The Second Affiliated Hospital, Guangzhou University of Chinese Medicine, Guangzhou, China; ^2^ Xiaorong Luo’s National Renowned Expert Inheritance Studio, Guangdong Provincial Hospital of Chinese Medicine, Guangzhou, China

**Keywords:** macrolide-unresponsive *Mycoplasma pneumoniae* pneumonia (MUMPP), macrolide-sensitive *Mycoplasma pneumoniae* pneumonia (MSMP), clinical characteristics, predictive indictors, early and appropriate treatment

## Abstract

**Introduction:**

Macrolide-unresponsive *Mycoplasma pneumoniae* pneumonia (MUMPP) cases have been rapidly increasing. The primary reason for this increased incidence is the pathogen's acquisition of resistance through mutations in 23S rRNA genes. Due to the unfeasibility of testing for macrolide susceptibility at the time of admission, this study aimed to assess the clinical features of pediatric MUMPP, using insights from laboratory tests and patterns of chest radiographic resolution.

**Material and methods:**

We conducted a retrospective review of 161 patients with *M. pneumoniae* pneumonia (MPP) between January 2023 and December 2023. These patients were categorized into two groups based on their responsiveness to macrolides: 72 patients were in the MUMPP group, and 89 patients were in the macrolide-sensitive *Mycoplasma pneumoniae* pneumonia (MSMP) group.

**Results:**

MUMPP patients experienced a longer duration of fever and hospital stay. A higher proportion of MUMPP patients had shortness of breath, transcutaneous blood oxygen saturation (SpO_2_) lower than 94%, bilateral lobar infiltrates, lobar pneumonia and pleural effusion. The serum level of serum ferritin (SF), interleukin-6(IL-6), D-dimer, lactate dehydrogenase to albumin rate (LAR), and neutrophil to lymphocyte rate (NLR) were higher in MUMPP group.

**Conclusions:**

Our findings revealed that patients with MUMPP exhibit more severe initial radiographic indicators and clinical course compared to those with MSMP. Therefore, it is crucial to promptly administer alternative therapeutic agents besides macrolides for the management of MUMPP.

## 1 Introduction

*Mycoplasma pneumoniae* pneumonia (MPP) is a type of the community-acquired pneumonia (CAP), with *M. pneumoniae* being the primary cause of lower respiratory tract infection (LRTI) in children. MPP typically manifests as a benign, self-limiting condition associated with a positive prognosis. However, infrequent complications in other organs can pose significant health risks. Globally, M. pneumoniae epidemics occur every 3–7 years, with varying incidence rates (1–5). Recent report indicates a sharp rise in *M. pneumoniae* cases in several regions of China since June 2023 (6). Meanwhile, an increase of the incidence of infections with *M. pneumoniae* has also observed in 2023/2024 in Europe (7).These *M. pneumoniae* infection often leads to more severe clinical symptoms, which can not only impact the health of patients but also inflicts an economic burden on their families, often correlating with the severity of the infection and the effectiveness of antibiotic treatment.

After infection, *M. pneumoniae* adheres to the host's respiratory epithelium and produces numerous cytotoxic proteins, protecting itself from removal by mucociliary escalator mechanisms while damaging the host's pseudostratified epithelium (8). A recent study demonstrated that Club Cell Secretory Protein (CC16) plays an important role in the airway remodeling and pulmonary epithelium damage during respiratory infection with *M. pneumoniae* (9). Reports also indicate that the pathogenesis of *M. pneumoniae* infection is closely related to the stimulation of macrophages by this pathogen via toll-like receptors, which release immunomodulatory and inflammatory cytokines and chemokines (10). The severity of *M. pneumoniae* infection is related to the host immune system's response to the infection (11).

Conventionally, macrolides have been the preferred choice of antimicrobials for treating *M. pneumoniae* infections in children (11). However, recent observations indicate that despite treatment with macrolides for over 7 days, some cases still exhibit worsening clinical symptoms like persistent fever and aggravated lung imaging, leading to refractory MPP (RMPP) (12). Although macrolides are effective in treating RMPP, alternative treatments are required in cases of prolonged fever and hospitalization. The increasing prevalence of macrolide-resistant *M. pneumoniae* compromises treatment effectiveness. Additionally, excessive immune response, co-infections, and host conditions contribute to lung injury in MPP, further affecting the efficacy of macrolide therapy (13, 14). Timely and appropriate treatment is crucial to prevent complications like necrotizing pneumonia, bronchiolitis obliterans, thrombosis, and even death (15). Therefore, predicting macrolide responsiveness and susceptibility in the early stages of *M. pneumoniae* infection is essential.

Predicting treatment response at admission has recently become increasingly challenging. Although distinguishing between RMPP and MSMP has recently garnered attention, obtaining records of the gradually worsening syndrome or chest x-ray is difficult. Additionally, clinicians often switch treatments within 7 days of initial macrolide use for MPP patients. To confirm macrolide resistance, the drug resistance gene detection is required. However, drug resistance gene detection is not a routine test item and can be costly. Based on an actual treatment course, macrolide-unresponsive Mycoplasma pneumoniae pneumonia (MUMPP) is characterized by a persistent fever of 38.0℃ or higher, lasting for 72 h or more following macrolide therapy. Despite its frequency in clinical settings, few studies have characterized this condition. Patients with MUMPP face a higher risk of progressing to RMPP and/or severe clinical outcomes compared to MSMP patients, making it a critical issue in clinical practice (16). Although some reports indicate that switching to secondary treatments does not improve therapeutic efficacy compared to prolonged macrolide use in children with MUMPP (17), early administration of tetracycline antibiotics may effectively prevent the progression of MUMPP to RMPP and/or mitigate severe clinical outcomes (16).

In this study, patients were categorized into two distinct groups based on the duration of fever after macrolide administration: MUMPP (persistent fever of ≥38.0°C for ≥72 h after macrolide administration) and MSMP (fever subsided within 3 days). This study sought to compare and identify the differences in clinical and radiological characteristics between these two groups. Additionally, we evaluated potential markers such as IL-6, SF, D-dimer, and LDH for predicting MUMPP. This distinction may assist physicians in administering safe and effective treatment strategies, enhancing the efficacy of early-stage MPP management. Ultimately, this research aims to determine the distinguishing features of MUMPP from MSMP, thereby contributing to the advancement of MPP management strategies.

## 2 Materials and methods

### 2.1 Subjects and study design

General information: We conducted a retrospective analysis of the medical records of patients aged ≤18 years admitted to our department from January 1, 2023, to December 31, 2023, and diagnosed with MPP.

Inclusion criteria: (1) complete and comprehensive medical records; (2) hospitalized patients under 18 years of age; (3) Patients exhibiting signs and symptoms of CAP, including fever, acute respiratory symptoms (cough, tachypnoea, difficult breathing), or both, along with the presence of new infiltrates or consolidation on chest radiography; (4) positive result for *M. pneumoniae*. The diagnosis of *M. pneumoniae* infection was confirmed if any or all of the following conditions were met: (i) positive serologic tests, with the specific IgM titers against *M. pneumoniae* ≥1:160 or a four-fold or greater increase in IgM or IgG (or both) antibody titers between the acute and convalescent stages; (ii) Positive PCR or real-time PCR results for *M. pneumoniae* using nasopharyngeal aspiration or sputum samples.

Based on macrolide sensitivity, the MPP patients were divided into two groups: MUMPP and MSMP. MUMPP cases were clinically defined as those with persistent fever ≥38.0°C for at least ≥72 h after macrolide treatment. Conversely, MSMP cases were those whose fever subsided within 72 h after macrolide administration.

Exclusion criteria included: (1) patients with clinical symptoms and radiologic findings inconsistent with pneumonia, despite positive *M. pneumoniae* IgM; (2) patients who switched treatment within 72 h of the initial macrolide antibiotic administration; (3) patients with immunodeficiency disease, chronic pulmonary disease, kidney or liver disease, neoplasms, primary ciliary dyskinesia, cystic fibrosis, cardiovascular disease or malabsorption syndromes; (4) patients in the late stages of MPP.

### 2.2 Data collection

Demographic and clinical data were collected from medical histories, encompassing age, sex and BMI. Clinical characteristics included the duration and onset time of fever, catarrhal syndrome, hospital stay, transcutaneous blood oxygen saturation (SpO_2_), extrapulmonary complications and administered treatments. Upon admission, blood samples were assessed for total and differential cell counts, as well as serum levels of white blood cell (WBC), C-reactive protein (CRP); interleukin-6 (IL-6), serum ferritin (SF); erythrocyte sedimentation rate (ESR); aspartate aminotransferase (AST); alanine aminotransferase (ALT), lactate dehydrogenase (LDH); creatine kinase (CK); phosphocreatine kinase isoenzyme (CK-MB) and D-dimer. Additionally, the rates of certain serum indicators, including neutrophil to lymphocyte rate (NLR); platelet to lymphocyte rate (PLR); monocyte to lymphocyte rate (MLR); lymphocyte to CRP rate (LCR), and LDH to albumin rate (LAR), were evaluated.

Chest radiographs were conducted when clinically indicated, allowing for the detection of bronchopneumonia or lobar pneumonia, with or without pleural effusion and atelectasis. *M. pneumoniae* infection was diagnosed using DNA extracted from oropharyngeal swabs. Acute IgM serology and/or elevated IgG titers in serum were evaluated by using a commercial test kit. A comparative analysis was performed to assess the differences in the aforementioned indicators between the MUMPP and MSMP groups. The majority of clinical information and chest radiographs reported in this study were reviewed by pediatric pulmonary specialists.

### 2.3 Data analysis

Categorical variables were summarized in terms of frequency and expressed as percentages, while serial data were analyzed in two ways. When the assumption of normal distribution was met, serial data were analyzed using the mean and standard deviation. Conversely, when the data was not normally distributed, the median and interquartile range were used to represent the serial data.

Continuous variables were compared using the paired *t*-test (or Wilcoxon test, as applicable) for normally distributed data, whereas the Mann-Whitney test was used for non-normally distributed data. Categorical variables between the two groups were compared using the Chi-Square test, with Fisher's exact test being preferred in instances where at least one expected frequency was less than 5. The significant variables were further described using the receiver operating characteristic (ROC) curve, and optimal cut-off points were determined using the area under the curve (AUC). All statistical analyses were performed using SPSS software (version 22) and Prism GraphPad (version 10), with a significance level set at *p* < 0.05.

## 3 Results

### 3.1 Subject's demographics

A total of 161 patients diagnosed with *M. pneumonia* and treated in our hospital's pediatrics department were included in the study. Table 1 outlines the patients' demographic details. Out of these, 48.8% (78) patients were male. The median age was 7.16 years (±2.57), with a BMI of 15.22 kg/m^2^ (±2.28), and a median duration of fever (from the initial onset) of 8.93 days (±4.02). The presence of *M. pneumoniae* was detected throughout 2023, with a significant peak observed in the fourth quarter. According to the inclusion criteria, the patients were further categorized into two groups: 72 patients belonged to the MUMPP group, whereas the remaining 89 patients were in the MSMP group. The average hospital stay was 6.12 days (±2.39) and the average duration of macrolide treatment was 5.79 days (±2.04).

**Table 1:** Subject's demographics.

Variables	No. (%) or mean ± SD
Total number of patients	161
Age, years	7.16 ± 2.57
Male sex	78 (48.8%)
Time of onset (the fourth quarter)	96 (60%)
BMI [kg/m^2^]	15.22 ± 2.28
MUMMP/MSMP	72/89
Total duration of fever (days)	8.93 ± 4.02
the treatment duration with macrolides (days)	5.79 ± 2.04
Hospital days (days)	6.12 ± 2.39

All data are presented as either the number (%) or mean (±SD).

MUMPP, macrolide-unresponsive *M. pneumoniae* pneumonia; MSMP, macrolide-sensitive *M. pneumoniae* pneumonia; BMI, body mass index; SD, standard deviation.

### 3.2 Comparison of clinical characteristics between the two groups

The clinical differences between MUMPP and MSMP children are outlined in Table 2. Both groups exhibited no significant difference in terms of age, gender, BMI, onset time, or catarrhal syndrome. However, the MUMPP group had a significantly longer median fever duration before admission, standing at 7 (5.25, 9.75) days, compared to the MSMP group's 6 (5, 8) days (*p* = 0.0118). Additionally, the median length of stay for MUMPP patients was significantly longer, standing at 6.5 (5, 8) days, in contrast to the 5 (4, 6) days for the MSMP group (*p* < 0.001).

**Table 2:** Comparison of clinical characteristics between the two groups.

Category	MUMMP		MSMP		*χ*^2^/t/z	*p*
Number of subjects	72	Number of actual responses	89	Number of actual responses		
Age [*y*, ($\bar{x}\pm s$)]	7.33 ± 2.58	72	7.03 ± 2.57	89	0.7327	0.4308
Male sex, *n* (%)	31 (43.06)	72	47 (52.81)	89	1.406	0.2673
BMI [kg/m^2^, ($\bar{x}\pm s$)]	14.93 ± 1.97	71	15.45 ± 2.48	88	1.462	0.1458
Time of onset (the fourth quarter), *n* (%)	40 (55.56)	72	56 (62.92)	89	0.897	0.4195
Catarrhal syndrome, *n* (%)	19 (26.39)	72	25 (28.09)	89	0.05799	0.86
duration of fever before admission [*d*, M (IQR)]	7 (5.25,9.75)	72	6 (5,8)	88	2.915	0.0118
Hospital days [d, M(IQR)]	6.5 (5,8)	72	5 (4,6)	89	4.382	<0.001
Shortness of breath, *n* (%)	17 (23.61)	72	9 (10.11)	89	5.356	0.0207
SpO_2_ < 94%, *n* (%)	21 (29.17)	72	14 (15.73)	89	4.223	0.0399
SMPP, *n* (%)	38 (52.78)	72	31 (34.83）	89	5.234	0.0256
Develop to SMPP after admission, n (%)	12 (16.67)	72	3 (3.37)	89	8.328	0.0039
23S rRNA gene mutation, n (%)	35 (63.64)	55	19 (52.78)	36	1.063	0.3836
Receipt of antibiotics prior to admission within 5 d, *n* (%)	72 (100)	72	76 (85.39)	89	3.382	0.0006
Receipt of macrolides before admission, *n* (%)	62 (86.11)	72	56 (62.92)	89	10.93	0.0011
Duration from onset to the first receipt with macrolides [d, M (IQR)]	4 (2,4)	71	6 (5,7)	88	4.437	<0.001
Receipt of penicillin before admission, *n* (%)	4 (5.56)	72	7 (7.87)	89	0.3335	0.7557
Receipt of cephalosporins before admission, *n* (%)	33 (45.83)	72	41 (46.07)	89	0.0296	0.999
Receipt of antiviral drugs before admission, *n* (%)	6 (8.33)	72	11 (12.36)	89	0.6832	0.451
Inpatient combination with Cephalosporins, *n* (%)	14 (19.44)	72	19 (21.35)	89	0.08853	0.8455
Inpatient combination with Penicillin, *n* (%)	3 (4.17)	72	3 (3.37)	89	0.07027	0.9999
Inpatient antiviral drugs, *n* (%)	2 (2.78)	72	6 (6.74)	89	1.324	0.2991
Oxygen support, *n* (%)	31 (43.06)	72	22 (24.72)	89	6.06	0.0181
High-flow oxygen support, *n* (%)	12 (16.67)	72	5 (5.62)	89	5.858	0.0368
Flexible bronchoscopy, *n* (%)	49 (68.06)	72	28 (31.46)	89	4.622	<0.0001
Change the antibiotic to DXC, *n* (%)	51 (70.83)	72	18 (20.22)	89	6.452	<0.0001
TTD after initial DXY treatment [h, M (IQR)]	24 (12,48)	51	6 (0,24)	18	2.322	0.0096
Treatment added on CST, *n* (%)	4 (5.56)	72	3 (3.37)	89	0.4568	0.7013

All data are presented as either the number (%), mean (± SD) or mean (IQR). For items with missing responses, the number of actual responses was entered in the column on the right.

MUMPP, macrolide-unresponsive *M. pneumoniae* pneumonia; MSMP, macrolide-sensitive *M. pneumoniae* pneumonia; SpO_2_, transcutaneous blood oxygen saturation; DXY, doxycycline; TTD, time to defervescence; CST, corticosteroids.

Notably, 100% of MUMPP patients received antibiotics within 5 days prior to admission, compared to 85.39% in the MSMP group (*p* = 0.0006) (Table 2). While the difference in the proportion of patients treated with macrolides before admission was statistically significant between the two groups, no significant differences were observed in the proportions of patients receiving cephalosporins or penicillin. Furthermore, MUMPP patients experienced a shorter duration from onset to the first administration of macrolides compared to the MSMP group. Compared to antibiotics, antiviral drugs showed a lower administration rate before admission, with no significant difference between the two groups.

During hospitalization, the combination of antibiotics and antiviral drugs was not significantly different between the two groups. However, the MUMPP group exhibited a higher frequency of SpO_2_ < 94% and SMPP, which require more frequent use of flexible bronchoscopy and oxygen support, particularly heated and humidified high-flow oxygen. Among these SMPP patients, MUMPP group had a higher rate of transitioning to severe MPP(SMPP) after admission compared to MSMP patients (*p* < 0.01).

In this study, the primary secondary treatment measures included the addition of corticosteroids (CST) or switching to doxycycline as the antibiotic. Notably, the MUMPP group had a significantly higher rate of switching to doxycycline (*p* < 0.05). Following doxycycline therapy, a median time to defervescence (TTD) of 24 h (interquartile range 24–48) in all patients, with the MUMPP group experiencing a longer median duration of 24 h compared to 6 h in the MSMP group. The addition of CST was observed in only a small proportion of cases, with no significant difference between the two groups.

In the MUMPP group, with the exception of prolonged macrolide treatment (PMC), the primary therapeutic approach was switched to doxycycline (DXC). We categorized the MUMPP patients into two groups: the PMC group and the DXC group. Within the DXC group, patients were further divided based on the duration of macrolide treatment prior to DXC administration: those with less than 5 days and those with more than 5 days of macrolide treatment. Our findings indicate that early administration of DXC can improve both the duration of fever and hospital stay, with a statistically significant difference observed in the duration of fever. (*p* < 0.05) (Table 3).

**Table 3:** Efficacy of different therapy for MUMPP.

Category	PMC (*n* = 21)	DXC(<5d, *n* = 26)	DXC(≥5d, *n* = 25)	*P*
Duration of fever [d, M (IQR)]	11 (9,14)	9 (7.11)	11 (9,13)	<0.05
Hospital stay [d, M (IQR)]	7 (5,10)	6 (6.8)	6 (5,8)	>0.05

All data are presented as mean (IQR).

PMC, prolonged macrolide; DXC, doxycycline.

### 3.3 Extrapulmonary manifestations

Different extrapulmonary manifestation in patients with *M. pneumoniae* and their relative distribution are shown in Table 4. Overall, 58 (36.6%) patients exhibited one or more extrapulmonary manifestation, with a notably higher prevalence in the MUMPP group, accounting for 39 (54.17%) cases, compared to the MSMP group with 19 (21.35%) cases. Notably, the MUMPP group exhibited a greater frequency of gastric syndrome than the MSMP group in this study. While there were no significant differences in skin and mucosal, hepatobiliary system, and cardiovascular involvement between the two groups, these manifestations were more common in the MUMPP group.

**Table 4:** Extrapulmonary manifestations.

Category	MUMMP	MSMP	χ^2^/t/z	*p*
Any manifestation, *n* (%)	39 (54.17)	19 (21.35)	18.6	<0.0001
Skin and mucosa rash, *n* (%)	5 (6.94)	4 (4.49)	0.4527	0.515
Digestive system syndrome, *n* (%)	18 (25)	7 (7.87)	2.985	0.004
Liver enzyme elevation, *n* (%)	3 (4.17)	0 (0)	3.779	0.0874
Myocardial enzyme elevation, *n* (%)	9 (12.5)	5 (5.62)	2.374	0.1616
Anemia, *n* (%)	8 (11.11)	3 (3.06)	4.44	0.0551

All data are presented as the number (%).

MUMPP, macrolide-unresponsive *M. pneumoniae* pneumonia; MSMP, macrolide-sensitive *M. pneumoniae* pneumonia.

### 3.4 Comparison of serum markers at admission between the two groups

The serum levels of inflammatory cytokines were examined in the children with MUMPP group and MSMP. The findings revealed significantly elevated levels of NEUT% (67.5 vs. 62.4, *p* < 0.05), CRP (15.79 vs. 11.3 mg/L, *p* < 0.05), PCT (0.19 vs. 0.115 ng/ml, *p* < 0.05), IL-6 (21 vs. 11.9 pg/ml, *p* < 0.05), SF (176.4 vs. 122.1 ug/L, *p* < 0.05), and NLR (3.382 vs. 2.611, *p* < 0.05) in the MUMPP patients compared to the MSMP group.

Moreover, we observed significant difference in serval systemic indicators, as detailed in Table 5. Compared to the MSMP group, the MUMPP group showed significantly higher median values of serum total LDH (343.1 ± 146.6 vs. 291. 7 ± 74.74 IU/L, *p* = 0.0051), AST (35.6 vs. 30.85 IU/L, *p* < 0.05), albumin (39.56 vs. 40.46 g/L, *p* < 0.05), LAR (8.784 vs. 7.003, *p* < 0.05), CK (169.1 vs. 118.9, *p* < 0.05), and D-dimer (0.73 vs. 0.51 mg/L, *p* < 0.05). However, no significant differences were noted in WBC, neutrophil counts, platelet, EOS, monocytes, ESR, ALT, and CK-MB between the two groups.

**Table 5:** Comparison of serum markers at admission between the two groups.

Categories	MUMMP		MSMP		χ^2^/t/z	*p*
Number of subjects	72	Number of actual responses	89	Number of actual responses		
WBC [×10^9^, ($\bar{x}\pm s$)]	7.35 ± 3.04	71	7.5 ± 2.71	89	0.3236	0.4172
Neutrophil [%, M (IQR)]	67.5 (61.8, 74.4)	71	62.4 (57.05, 69.05)	89	3.433	0.0013
Neutrophil counts [×10^9^, M (IQR)]	4.51 (3.35, 5.91)	68	4.11 (3.19, 5.56)	81	1.166	0.277
Lymphocyte counts [×10^9^, M (IQR)]	1.51 (1.24, 1.94)	68	1.75 (0.7, 6.69)	81	2.437	0.0194
NLR ($\bar{x}\pm s$)	3.38 ± 1.9	68	2.61 ± 1.77	81	2.563	0.0006
Monocyte counts [×10^9^, M (IQR)]	0.48 (0.31, 0.61)	68	0.53 (0.38, 0.72)	80	1.332	0.0828
Platelet counts [×10^9^, M (IQR)]	269 (239, 356)	69	279 (236, 361)	89	0.6073	0.5003
Eosinophil counts [×10^9^, M (IQR)]	0.08 (0.03, 0.23)	69	0.1 (0.05, 0.23)	88	1.62	0.1654
PLR ($\bar{x}\pm s$)	201.1 ± 85.52	68	168.3 ± 67.79	80	2.602	0.0205
MLR ($\bar{x}\pm s$)	0.31 ± 0.14	68	0.29 ± 0.12	80	0.9934	0.4591
LCR ($\bar{x}\pm s$)	0.85 ± 4.12	71	0.72 ± 3.14	84	0.2176	0.2169
CRP [mg/L, ($\bar{x}\pm s$)]	21.5 ± 21.07	72	14.28 ± 12.76	85	2.64	0.0338
Procalcitonin [ng/ml, M (IQR)]	0.19 (0.09, 0.4)	71	0.12 (0.07, 0.20)	86	0.5182	0.0046
ESR [mm/h, M (IQR)]	59 (43, 75.75)	72	57.5 (40.5, 72.25)	82	0.5294	0.5885
IL-6 [pg/ml, M (IQR)]	21 (10.5, 27.1)	45	11.9 (6.3, 21.15)	57	2.795	0.0011
SF [ug/L, M (IQR)]	176.4 (123.9, 241.4)	31	122.1 (82.56, 178)	41	2.59	0.0035
D-Dimer [mg/L, M (IQR)]	0.73 (0.51, 1.22)	55	0.51 (0.31, 0.735)	69	1.798	0.0002
ALT [U/L, M (IQR)]	13.5 (10, 18)	72	12 (10,14)	88	1.488	0.0775
AST [U/L, $\bar{x}\pm s$]	35.6 ± 14.61	72	30.85 ± 8.788	85	2.513	0.013
Albumin [g/L, $\bar{x}\pm s$]	39.56 ± 3.38	72	40.46 ± 2.189	83	1.997	0.0476
CK [U/L, $\bar{x}\pm s$]	169.1 ± 183.2	71	118.9 ± 132	86	1.993	0.0481
CKMB [U/L, $\bar{x}\pm s$]	20.86 ± 6.471	70	20.21 ± 6.183	86	0.3409	0.7337
LDH [U/L, $\bar{x}\pm s$]	343.1 ± 146.6	71	291.7 ± 74.74	86	2.84	0.0051
LAR ($\bar{x}\pm s$)	8.784 ± 4.69	72	7.003 ± 1.923	83	3.168	0.0019

All data are presented as either mean (± SD) or mean (IQR). For items with missing responses, the number of actual responses was entered in the column on the right.

MUMPP, macrolide-unresponsive *M. pneumoniae* pneumonia; MSMP, macrolide-sensitive *M. pneumoniae* pneumonia; WBC, white blood cell; CRP, C-reactive protein; IL-6, interleukin-6, SF, serum ferritin; ESR, erythrocyte sedimentation rate; AST, aspartate aminotransferase; ALT, alanine aminotransferase, LDH, lactate dehydrogenase; CK, creatine kinase; CK-MB, phosphocreatine kinase isoenzyme; NLR, neutrophil to lymphocyte rate; PLR, platelet to lymphocyte rate; MLR, monocyte to lymphocyte rate; LCR, lymphocyte to CRP rate; LAR, LDH to albumin rate.

**Figure 1:**
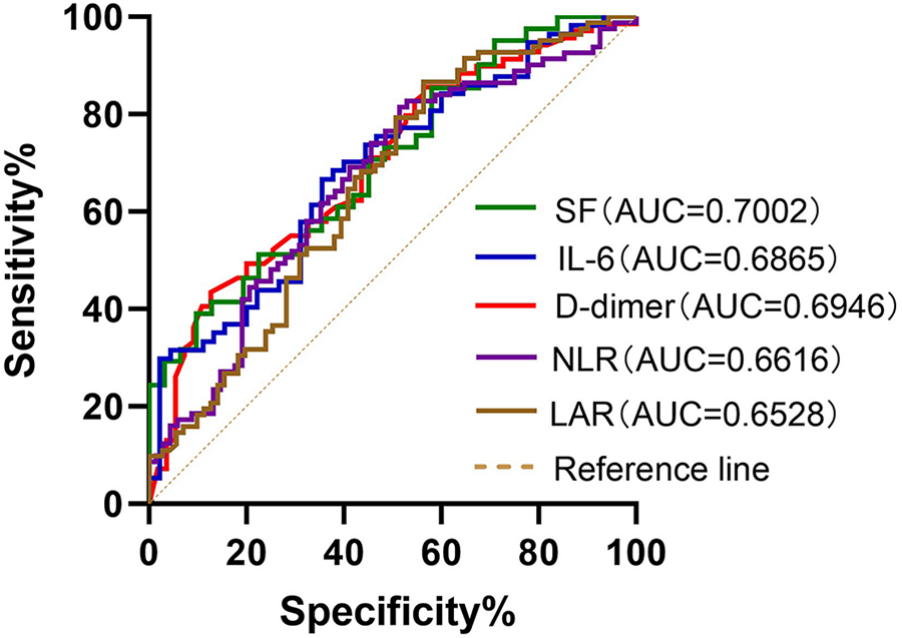
Receiver operating characteristic (ROC) curves of SF, IL-6, D-dimer, NLR and LAR for predicting MUMPP. SF had better predictive ability for differentiation of MUMPP. SF, serum ferritin; IL-6, interleukin-6; NLR, neutrophil to lymphocyte rate; LAR, lactate dehydrogenase to albumin rate; MUMPP, Macrolide-unresponsive *Mycoplasma pneumoniae* pneumonia; AUC, area under the curve.

ROC curves were generated to further evaluate the predictive value of these risk factors for MUMPP. The areas under the ROC curves for SF, IL-6, D-dimer, LAR, PCT, NLR, LDH and CRP were 0.7002, 0.6865, 0.6946, 0.6528, 0.631, 0.6616, 0.6218, and 0.5984 respectively (*p* < 0.01). Among these, SF exhibited the largest area under the ROC curve, indicating its highest predictive value for MUMPP in MPP patients. At the specified cut-off value, SF showed high specificity (90.32%) but relatively low sensitivity (39.02%). The cut-off values were: SF at 104.1 ug/L (39.02% sensitivity, 90.32% specificity), NLR was 3.212 (81.48% sensitivity, 48.53% specificity), LAR was 8.476 (86.59% sensitivity, 43.66% specificity), IL-6 was 16.81 pg/ml (66.67% sensitivity, 64.44% specificity), D-dimer was 0.435 mg/L (43.348% sensitivity, 87.27% specificity), PCT was 0.2151 ng/ml (76.74% sensitivity, 47.89% specificity), LDH was 291 U/L (60.47% sensitivity, 60.56% specificity) and CRP was 26.21 mg/L (89.41% sensitivity, 30.56% specificity). As demonstrated above, SF, IL-6, D-dimer, LAR, and NLR exhibited the highest areas under the ROC (Figure 1), meaning that these were the best indicators for predicting MUMPP.

### 3.5 Chest imaging

The features of chest radiographs obtained at admission and the distinguishing characteristics of each patient group are summarized in Table 6. The proportion of patients with bronchopneumonia was comparable in the MUMPP group (12.5%) and the MSMP group (11.24%). Compared with the MSMP group, the MUMPP group showed no significant difference in consolidation. However, the MUMPP group exhibited a significantly higher likelihood of unilateral lobar infiltrates and a lower tendency towards bilateral lobar infiltrates. Specifically, the percentage of patients with lobar pneumonia alone was significantly higher in the MUMPP group (55.56%) compared to the MSMP group (44.44%) (*p* < 0.01). Pleural effusion was observed in a total of 25 patients (15.23%), with a significantly greater proportion noted in the MUMPP group (22.22%) compared to the MSMP group (10.11%). Furthermore, bronchiolitis and atelectasis were detected in only a minor fraction of patients, showing no significant differences between the two groups (*p* > 0.05).

**Table 6:** Chest imaging.

Category	MUMMP	MSMP	χ^2^/t/z	*P*
Bronchopneumonia, *n* (%)	9 (12.5)	10 (11.24)	0.2472	0.8112
Unilateral lobar infiltrates, *n* (%)	30 (41.67)	52 (58.43)	4.474	0.04
Bilateral lobar infiltrates, *n* (%)	33 (45.83)	21 (23.6)	8.83	0.003
Atelectasis, *n* (%)	5 (6.94)	2 (2.25)	2.112	0.2439
Bronchiolitis, *n* (%)	2 (2.78)	2 (2.25)	0.2151	0.8279
Lobar pneumonia, *n* (%)	35 (55.56)	28 (44.44)	4.915	0.0266
Pleural effusion, *n* (%)	16 (22.22)	9 (10.11)	4.45	0.0481

All data are presented as the number (%).

MUMPP, macrolide-unresponsive *M. pneumoniae* pneumonia; MSMP, macrolide-sensitive *M. pneumoniae* pneumonia.

## 4 Discussion

The recent surge in MUMPP incidence, particularly in children, is concerning and linked to macrolides resistant *Mycoplasma pneumoniae* strains infection and potentially life-threatening conditions (14, 18). Beyond increased bacterial load, an excessive host immune response among patients with MUMPP may lead to lung injury (13, 14). Without proper treatment, these patients may experience poor outcomes and prolonged disease courses, with numerous complications during the acute stage and potential long-term sequelae such as post-infectious bronchiolitis obliterans, bronchial asthma, unilateral transparent lung, and bronchiectasis (3, 19). Therefore, analyzing predictive factors for MUMPP and establishing early detection and timely treatment is crucial to prevent complications and sequelae.

Consistent with other studies, the median age of MPP patients was 7 years, with no statistically significant gender difference. *M. pneumoniae* incidence peaks in the summer and fall seasons in America (20) and between August and January in China (21). Similarly, more than half of MPP patients in our study were detected in the fourth quarter. Longer median hospital stay (8.97 days) often burdens medical resources and clinical staff, especially during epidemics. The clinical symptoms associated with *M. pneumoniae* CAP in multivariable analysis resemble those of respiratory viral illnesses (22), making it challenging to distinguish *M. pneumoniae* from other pathogens. However, related syndromes such as catarrhal syndrome were less common in our study (27.3%). The median BMI was lower in the MUMPP group, suggesting that smaller individuals may be less sensitive to macrolides and prone to severe syndromes. However, BMI showed no significant difference between the groups, highlighting the need for a larger patient sample in future studies.

Compared to the MSMP group, the MUMPP group exhibited a greater proportion of severe clinical syndromes, characterized by significantly longer fever duration, prolonged hospital stays, and more pronounced symptoms, including a higher percentage of shortness of breath (23.61%) and SpO_2_ < 94% (29.17%). Furthermore, the ratio of SMPP ones was significantly higher in the MUMPP group than in the MSMP group (52.78% vs. 34.83%; *p* < 0.05), consistent with prior research (23). Although no patients required ICU admission with mechanical ventilation, a larger proportion of MUMPP patients required oxygen therapy, particularly heated and humidified high-flow oxygen support.

*M. pneumoniae* is known to induce a wide range of extrapulmonary manifestations affecting nearly every organ system, potentially leading to more severe medical complications than pneumonia (24). In our study, the MUMPP group exhibited a significantly higher proportion of extrapulmonary manifestations compared to the MSMP group. Gastrointestinal symptoms were the most prevalent extrapulmonary manifestation, particularly in the MUMPP group. However, MP-associated myocarditis, encephalitis, and other extrapulmonary complications were not observed, possibly due to the limited number of MPP patients included. In clinical practice, the possibility of MUMPP should be considered when similar intrapulmonary and extrapulmonary manifestations are observed in children.

Following *M. pneumoniae* infection, disruptions in innate and adaptive immunity lead to excessive inflammation in the lungs and throughout the body (25). In turn, the cytokines and chemokines released during these hyperinflammatory responses further amplify the inflammatory cascade (26). In our study, we assessed several serological markers to predict MUMPP. Our analysis revealed that the MUMPP group had significantly higher levels of N%, IL-6, SF, CRP, PCT, AST, D-dimer, and LDH compared to the MSMP group. Additionally, the neutrophil-to-lymphocyte ratio (NLR) and LDH-to-albumin ratio (LAR) were significantly higher in the MUMPP group. These findings suggest that excessive inflammatory and immune responses play crucial roles in the pathogenesis of MUMPP, consistent with previous studies (27, 28).

Ferritin is induced by activated macrophages, which produce tumor necrosis factor (TNF)-*α* (29). Serum ferritin (SF) often exhibits a non-specific increase in various infectious or inflammatory disorders, and its level can effectively reflect the body's immune defense ability (30). Previous studies have reported that the SF level rises progressively in children with MPP as their disease worsens (31). In our study, ROC curve analysis revealed that SF could be used as a predictor of MUMPP. SF had the largest AUC (0.7002) among all serum markers. Its predictive value for MUMPP was 104.1ug/L, with high specificity (90.32%) but low sensitivity (39.022%).

*M. pneumoniae* infection and abnormal immune response lead to systemic inflammation, vascular endothelial injury, subcutaneous collagen exposure, and vasoconstriction. This disrupts the balance of blood coagulation and anticoagulation, resulting in a hypercoagulable state and elevated D-dimer levels. Elevated D-dimer levels in children with MPP are linked to hypercoagulability and vascular endothelial dysfunction, correlating with disease severity (32, 33). Consistent with previous studies (34), our research shows that D-dimer is a predictive factor for MUMPP. Elevated D-dimer levels, especially >0.435 mg/L, contribute to the early diagnosis of MUMPP.

Lactic dehydrogenase (LDH), a non-specific inflammatory biomarker of tissue damage, is present in all tissue cytoplasm. Its release into the serum is linked to cell dissolution or membrane damage, serving as an important indicator of infection severity and inflammatory disease (35, 36). Previous studies suggest that LDH is a predictor of severity and a marker of glucocorticoid therapy efficacy in MPP patients (28, 37, 38). In the present study, we observed elevated LDH and decreased albumin levels, with the LDH to albumin ratio (LAR) having a higher AUC than LDH alone (0.6528 vs. 0.6218). The LAR is widely used as an indicator of tissue damage, nutritional status, and systemic inflammatory response. Previous studies have reported high LDH and low albumin levels in SMPP patients, with LAR assisting clinicians in evaluating the progression of severe *M. pneumoniae* infection (39). Our study identified an LAR cut-off of 8.476 for predicting MUMPP, with 86.59% sensitivity and 43.66% specificity.

Neutrophils, which participate in the first line of defense against infection, play a crucial role in the immune response to *M. pneumoniae* (40). Their numbers increase in peripheral blood (41), bronchoalveolar lavage fluid (25), and lung tissue (42) after *M. pneumoniae* infection. However, excessive inflammation may cause lymphocytes apoptosis, thereby reduce their numbers (43). This phenomenon has been observed in MPP patients (44). The neutrophil-to-lymphocyte ratio (NLR) in peripheral blood is a simple, rapid and widely available indicator that has been reported to be associated with poor prognosis in idiopathic pulmonary fibrosis, chronic obstructive pulmonary disease (45), and COVID-2019 (46). Research indicates that NLR at admission can predict the prognosis of MPP (47). One retrospective study (48) concluded that an NLR >3.92 might be a valuable predictor value for RMPP in children over 6 years old. Our study also found NLR has predictive value for MUMPP, with an NLR >3.212 at admission indicating MUMPP with a sensitivity and specificity of 81.48% and 48.53%, respectively.

Despite observing some differences, certain indicators such as CRP, a commonly used marker for the early evaluation and identification of MPP with severe complications (33, 49), demonstrated low predictive ability for MUMPP due to their combined low specificity and sensitivity. This may be attributed to their nonspecific nature and reflection of whole-body inflammation levels.

These findings indicate that MUMPP exhibits more severe pulmonary inflammatory reaction or tissue damage. A robust cellular immune response leads to severe ciliary dysfunction, reduced airway immune function, and impaired ciliary mucus clearance. This results in large-scale infiltration, atelectasis, and mucus plug formation, hindering the recovery lung inflammation (49, 50). In a study of 393 hospitalized children with MPP, lobar or segmental consolidation was the most common radiological finding (37%) (51). Pneumonia involving two or more lobes is more common in macrolide-refractory MPP patients (52, 53). Our study found unilateral infiltrates more frequent in the MSMP group and bilateral infiltrates more frequent in the MUMPP group, with a higher incidence of lobar pneumonia in the MUMPP group, consistent with previous studies (23).

The early assessment of MPP severity through imaging strategies is crucial to prevent adverse outcomes in clinical practice. Kim et al. (2021) demonstrated that children with MPP and pleural effusion had more severe pneumonia lesions and poorer treatment outcomes, leading to prolonged resolution of lung abnormalities (54). Our study found a higher proportion of pleural effusion in the MUMPP group compared to the MSMP group. Radiologic findings of lobar pneumonia and pleural effusion in the MUMPP group suggest severe illness due to macrolide resistance, higher *M. pneumoniae* burden, severe host reactions, or other refractory response factors. Therefore, in *M. pneumoniae* patients, the possibility of an ensuing refractory response to macrolides should be considered if bilateral infiltrates, pleural effusion, or lobar pneumonia are detected on chest radiography during the initial hospitalization period.

Clinical practice guidelines recommend macrolide antibiotic treatment for patients with LRTIs compatible with atypical pathogens like *M. pneumoniae* (55). Physicians often prescribe macrolides without positive microbiology results (56). In our study, almost all patients received antibiotics prior to admission, with macrolides being the most frequent outpatient antibiotic. Despite receiving macrolides earlier than the MSMP group, MUMPP patients were more likely to progress to SMPP and require flexible bronchoscopy. Previous study also suggests that early macrolide treatment or resistance does not necessarily mitigate the clinical severity of MPP (57). Therefore, early macrolide treatment may not prevent severe MPP manifestations.

Secondary treatment agents like tetracyclines, fluoroquinolones, and corticosteroids have been reported to improve MUMPP prognosis. Doxycycline (DXY), a tetracycline, is effective against both macrolide-susceptible and -resistant strains (58). In our study, 70.83% of MUMPP patients switched to DXY, a significantly higher proportion than the MSMP group (*p* < 0.05). Patients who switched antibiotics tended to have severe symptoms, resulting in a longer TTD in the MUMPP group compared to the MSMP group (median 24 h vs. 6 h). However, the average TTD with DXY was significantly shorter than those with prolonged macrolide use (17). Notably, no patients received glucocorticoids when administered DXY, and previous studies have also reported lower glucocorticoid use in the DXY group compared to the azithromycin group (59).

Japanese guidelines recommend switching to a tetracycline antibiotic if defervescence is not achieved within 48 h of macrolide therapy (60). Early oral doxycycline treatment can quickly improve clinical symptoms and promote the resolution of pulmonary inflammation (58, 61). Furthermore, the early use of minocycline may offer better economic and clinical benefits, even without test results for drug resistance genes (16). Our study showed that early use of DXC could significantly improve the duration of fever. These findings underscore the importance of promptly initiating appropriate second-line agents in treating MUMPP.

Therefore, identifying the risk factors of MUMPP is of great significant, especially in settings without possibility of testing mutations in the 23S ribosomal RNA gene. Children with MUMPP were more likely to experience prolonged fever, intrapulmonary or extrapulmonary complications, severe lung imaging changes, and elevated inflammatory markers (SF, IL-6, D-dimer, LAR, NLR). If such manifestations are observed in children, MUMPP should be highly considered, and early use of secondary agents should be contemplated. The indicators included in our study are easily obtainable and conducive to clinical use.

## Data Availability

The original contributions presented in the study are included in the article/Supplementary Material, further inquiries can be directed to the corresponding author.
